# Effect of Harvest Period on the Proximate Composition and Functional and Sensory Properties of Gari Produced from Local and Improved Cassava* (Manihot esculenta)* Varieties

**DOI:** 10.1155/2018/6241035

**Published:** 2018-04-05

**Authors:** Alphonse Laya, Benoît Bargui Koubala, Habiba Kouninki, Elias Nchiwan Nukenine

**Affiliations:** ^1^Department of Life and Earth Sciences, Higher Teachers' Training College of Maroua, University of Maroua, P.O. Box 55, Maroua, Cameroon; ^2^Department of Biological Sciences, Faculty of Science, University of Maroua, P.O. Box 446, Maroua, Cameroon; ^3^Department of Chemistry, Faculty of Science, University of Maroua, P.O. Box 814, Maroua, Cameroon; ^4^Department of Biological Sciences, Faculty of Science, University of Ngaoundéré, P.O. Box 454, Ngaoundéré, Cameroon

## Abstract

This study is aimed at evaluating the proximate composition and functional and sensory characteristics of gari obtained from five cassava varieties (*EN*,* AD*,* TMS92/0326*,* TMS96/1414*, and* IRAD4115*). These cassavas were harvested during the dry season 12 months after planting (12MAP) and in the rainy season (15MAP). Results showed that the characteristics of gari varied significantly (*p* < 0.05) with the variety and the harvest period. Gari from* EN* cassava harvested at 12MAP had the highest total carbohydrates (78.07% dry weight), starch (61%), and proteins content, while gari from TMS* 96/1414* variety (12MAP) had high amino acids (10.25 mg/g) and phenolic compounds (9.31 mg/g) content. The gari from* IRAD4115* had the highest value of ash content (20.62 mg/g) at 12MAP. The soluble sugar content was high in the gari from cassava harvested at 12MAP while free cyanide reduced significantly in gari from cassava harvested at 12MAP. The water absorption capacity, swelling power, and bulk density were significantly (*p* < 0.05) high in the gari from* EN* cassava variety at 12MAP. Compared to commercial gari (3.30), gari from* EN* local cassava had the best overall acceptability (4.35) followed by those obtained from* TMS92/0326* and* TMS92/1414* varieties, respectively.

## 1. Introduction

Cassava roots yield more carbohydrates per hectare than cereal crops and can be grown at a considerably lower cost [1]. Cassava roots are a staple food that provides carbohydrates for more than 2 billion people in the tropics. However, cassava roots spoil quickly after harvest. In order to avoid this loss, they must be sold or processed into by-products after harvest. Generally, cassava and its products are poor in proteins. The deficiency in certain essential amino acids depends mostly on the varieties and geographical conditions. In order to enhance the nutritional quality of cassava, it is processed into fermented products such as gari. Gari is one of the most popular cassava products consumed in Africa, Southeast Asia, and Brazil [2]. In Africa, fermented foods and beverages are produced using fermentation. These products have been consumed for a long time because of their numerous nutritional values. In effect, lactic acid bacteria (LAB) isolated from these products have been proven to be good sources of antimicrobials and therapeutics, and are accepted as probiotics [3]. Fermented foods represent one-third of total food consumed by human beings [4]. Fermentation enhances the nutrient content of foods through the biosynthesis of vitamins, essential fatty acids, essential amino acids, and proteins and by improving protein quality and fibre digestibility [5–7]. It also enhances micronutrient bioavailability and aids in degrading antinutritional factors [8]. About 83% of the total cyanogenic glucosides (linamarin and lotaustralin) are detoxified during processing of cassava tuber into gari [9] and 98% of the cyanide is lost when gari is cooked into* eba* [10]. No detectable cyanide has been found in gari roasted with palm oil [11]. However acceptability of gari depends on the final texture and sensory attributes after processing [1]. Fermentation of cassava mash usually takes one to two days [12].

It has been reported that traditional gari contains a certain amount of residual cyanide. This is due to the tendency to shorten fermentation time in order to meet growing market demand [13]. That is why samples of gari with cyanide concentrations above 10 mg of HCN/kg are from areas where the cassava mash is fermented for less than 12 hours [14]. Halliday et al. [15] reported that the high initial moisture content and inappropriate storage container are the major factors that could encourage bacteria and fungi contamination and proliferation in gari during storage.

Gari is imported from neighbouring Nigeria or from the southern part of Cameroon to the far north region of Cameroon. In the continuous quest for solution to the problem of malnutrition in the far north region of Cameroon, improving nutritional quality and safety of local foods through better processing methods is recommended.

This work is aimed at producing gari from cassava roots of two local and three improved varieties harvested at two different growing periods.

## 2. Material and Methods

### 2.1. Trial Site and Experimental Design

The study was conducted in the far north region of Cameroon. The region is characterized by a transient equatorial climate with a long dry season (October to April) and short rainy season (May to September). The annual precipitation is 1000 mm and the mean annual temperature is 30°C. The soil is sandy and clayey.

The experimental field was a randomized complete block design with four repetitions. Each repetition measured 25 m^2^ with 25 cassava plants spaced at 1 m apart. Five varieties of (local and improved) cassava (*Manihot esculenta* Crantz) were considered in this study. The improved varieties were TMS92/0326 and TMS96/1414 from IITA (International Institute of Tropical Agriculture) and IRAD4115 from IRAD (Institut de Recherche Agricole pour le Developpement) in Adamawa region. The two local varieties,* EN *(red, sweet variety) and* AD *(red, bitter variety), were highly appreciated by the population in the far north and adamawa regions, respectively. After planting, all cassava varieties were grown for 12 or 15 months. Their storage roots were harvested in May (dry season) and August (rainy season), respectively.

### 2.2. Cassava Processing into Gari

The preparation of the gari was done following the method described by Agbor-Egbe and Mbome [12] and Amamgbo et al. [16]. Storage roots of the local and improved cassava varieties were harvested, cleaned, peeled, washed, and grated manually with a grater (Ø = 2 mm). The resulting mash was packed in a muslin tissue which was tied with sewing cotton. Then, mash was dewatered by placing the muslin tissue between a set of thick and long wooden poles arranged beneath and on top such that the ends were strongly fastened together with ropes. The mash was allowed to ferment for two days (48 h) under ambient conditions. The fermented mash was sieved to remove fibrous materials and then garified in a shallow pot with the addition of a small quantity of palm oil (10 ml/200 g) in order to obtain yellow gari. The different garis were obtained after 20 minutes of dry roasting at 80°C–90°C. The garis were then weighed and packed in polyethylene bags and labelled according to the cassava variety used. The gari yield was calculated as described by Sobowale et al. [17].

### 2.3. Determination of the Proximate Composition of Cassava Gari

The dry matter of the different cassava (*Manihot esculenta *Crantz) root and gari was determined using the standard AOAC [18] method. Slurries (10% dry matter) of all samples were made and their pH values were measured using a pH meter (HI 8424 Microcomputer Hanna instruments). The ash content of the samples was determined using standard AOAC [18] method. The titratable acidity of the gari was determined by titration with NaOH 0.01 N [18]. Values were expressed in equivalent gram acetic acid per 100 g of sample.

The total protein content of the root and in the different gari was determined using acetyl acetone/formaldehyde method proposed by Devani et al. [19]. Samples were first mineralized [20] and the nitrogen content of the mineralization was evaluated after a reaction with ammonia (NH_3_) and acetyl acetone/formaldehyde. A conversion factor of 6.25 was used to determine the protein content of the samples. The Ninhydrin colorimetric method described by Michel [21] was used to evaluate the free amino acid content of the samples.

Free sugars and carbohydrates content of the roots and in the different gari were determined by Orcinol colorimetric method [22]. Free sugars were obtained after stirring dried sample in an 80% ethanol solution. As concerns the total carbohydrates, samples were first hydrolysed with 13 M H_2_SO_4_ (30 min, 25°C) and then heated at 100°C for two hours. Crude fibres and lipids content of the samples were determined according to the standard AOAC [18] method.

The starch content of the samples (cassava root and gari) was determined by the iodine spectrophotometric method as performed by Jarvis and Walker [23]. Results were expressed in gram per 100 g of sample. The total phenolic compounds were determined using the Folin-Ciocalteu reagent as described by Singleton et al. [24] and the results were expressed as equivalent mg of gallic acid per gram sample.

Cyanides were evaluated in the cassava roots and in the different gari sample using picrate colorimetric method proposed by de B. Baltha and Cereda [25] with some modifications. Cyanides were first extracted using a sodium phosphate buffer (0.1 M; pH 6). A standard curve was performed with KCN and results were expressed in terms of equivalent mg of HCN per gram of dry sample. All analyses were performed in triplicate. A commercial cassava gari coming from the South region of Cameroon was used as reference.

### 2.4. Evaluation of the Functional Properties of Cassava Gari

The bulk density of gari was determined using a measuring cylinder as performed by Adeleke and Odedeji [26].

The swelling kinetic was assessed as described by Koubala et al. [27] with slight modifications. About 500 mg of dried gari was introduced in a measuring cylinder (100 ml) where it was mixed with 50 ml of distilled water. The gari was allowed to hydrate for 60 minutes at room temperature and its volume recorded from that time till equilibrium.

The water absorption capacity (WAC) was assessed according to the method described by Koubala et al. [27]. For WAC, 200 mg of the sample was introduced in a conical flask containing 10 ml of distilled water. The sample was soaked, stirred, and left 60 minutes at room temperature (25–30°C). The slurry was put on a sintered glass filter to allow the water to leak. When water was no longer leaking for one hour, the sample was weighed, dried at 105°C (overnight), and weighed again. The WAC was expressed in terms of ml of water absorbed per gram of gari.

The swelling power (SP) was determined based on the method of Hung et al. [28] with slight modifications. Measuring cylinder (10 ml) was filled with gari to the 2 ml mark and weighed. It was later made up to 10 ml with distilled water. The top of the cylinder was tightly covered and the content was mixed by inverting the cylinder. After each two minutes the cylinder was inverted again and left to stand for eight minutes. The final volume occupied by the gari was recorded.

In a tube, suspensions were made with gari sample in 5 ml of distilled water as carried out by Koubala et al. [27]. The tubes were heated at 90°C for one hour and allowed to cool overnight. Then, the gelation capacity was determined for each sample as the least gelation concentration. That is the concentration when the sample from the inverted test tube will not slip. The concentration of gari varied from 4% to 16%. All analyses were performed in triplicate. A commercial cassava gari coming from the South region of Cameroon was used as reference.

### 2.5. In-House Consumer Assessment

This assessment was conducted to determine consumer preferences and acceptability of the gari samples. Five-point hedonic scale as described by Nnanna et al. [29] was used with some modifications. A five-point hedonic scale is used (where 1 = dislike extremely, 1.5 = dislike very much, 2 = dislike moderately, 2.5 = dislike slightly, 3 = neither like nor dislike, 3.5 = like slightly, 4 = like moderately, 4.5 = like very much, and 5 = like extremely). The quality parameters assessed included the appearance or color, the taste (acidity and sweetness), the odor (aroma), the texture or mouthfeel and the overall acceptability. In-house consumer assessment was carried out within fifteen minutes of preparation. For this purpose, twenty trained panelists (students) coming from different regions of Cameroon, both males and females aged between 22 and 35, were involved. These panelists were usual consumers of gari and were chosen based on their ability to distinguish flavour, acidity, and sweetness in the gari sample. The gari samples were coded before being presented to the panelists who recorded their responses on the form provided in their slip. A commercial cassava gari coming from the South region of Cameroon was used as reference.

### 2.6. Statistical Analysis

The analysis was performed with Graph Pad (version 5.0; 2007). Results were presented as means ± standard deviation. The mean values were compared using independent sample Tukey's test, and the differences between them were determined by analysis of variance. As concerns in-house consumer assessment, Chi-square test was used to identify the determining factors and the overall acceptability of the panelists for the various gari samples. Tukey's multiple comparison test was used to determine the gari sample that was different from the others [30].

## 3. Results

In the present work, it is found that characteristics of the different processed gari varied significantly (*p* < 0.05) according to the harvest period and from one variety to another. Differences were also observed between gari from local and improved cassava variety.

### 3.1. Yield and Proximate Composition of Cassava Gari

**Figure 1:**
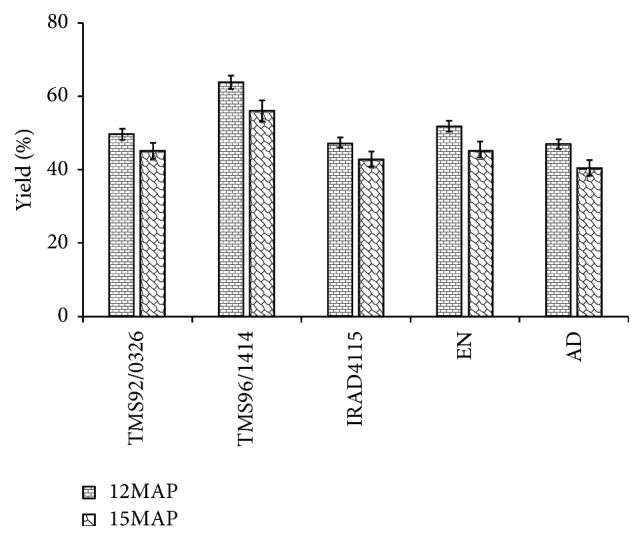
Effect of the harvest period (12 and 15 months after planting) on gari yield (g/100 g of wet mash). Garis are produced from local (*EN *and* AD*) and improved (*TMS92/0326, TMS96/1414, *and* IRAD4115*) cassava varieties harvested in dry season at 12 months and in rainy season at 15 months after planting (12MAP and 15MAP).

Results in Figure 1 show that gari produced from cassava root harvested 12 month after planting (12MAP) in the dry season showed the highest yield. Whatever the harvest period (12MAP or 15MAP), gari obtained from* TMS96/1414* cassava variety significantly (*p* < 0.05) exhibited the highest yield (63.72 g/100 g of wet mash). It is also noticed that the lowest gari yield was obtained with* AD *cassava (Figure 1). Generally the best gari yield was obtained with the improved cassava varieties.

Table 1 shows that the dry matter content of the different cassava gari was significantly (*p* < 0.05) similar (96-97%); but that of the commercial gari was significantly (*p* < 0.05) low (83.48%). The pH of the samples of gari fluctuated from 4.31 to 4.72. It is noticed that the pH of gari is significantly affected by the harvest period or age of the cassava root used for garification. Gari from cassava roots harvested at 12MAP exhibited the lowest pH values. This suggested the presence of an important quantity of organic acid in the gari. However, the commercial gari used as reference in our study recorded the highest pH value. The ash content of the cassava gari varied from 19.59 to 24.49 mg/g (dry weight). Gari from cassava roots harvested at 12MAP exhibited the lowest value of ash content (Table 1). The ash content of the commercial cassava gari was significantly (*p* < 0.05) similar to that of gari from cassava roots harvested at 12MAP in the dry season.

**Figure 2:**
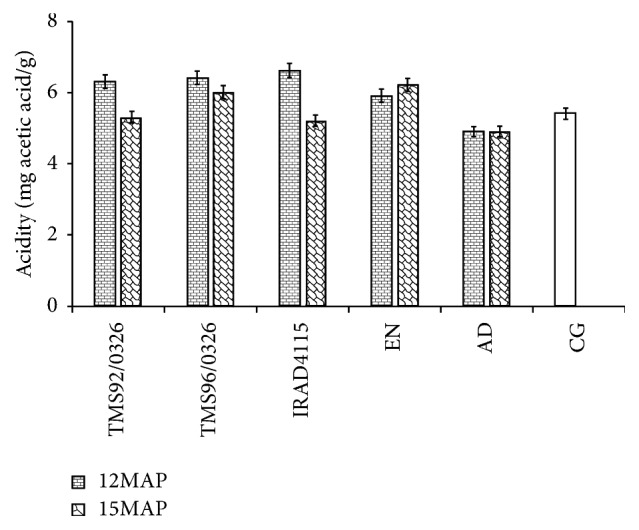
Influence of the harvest period (12 and 15 months after planting) on the titratable acidity (equivalent mg of acetic acid/g of dry weight) of cassava gari. Garis are produced from local (*EN *and* AD*) and improved (*TMS92/0326, TMS96/1414, *and* IRAD4115*) cassava varieties harvested in dry season at 12 months and in rainy season at 15 months after planting (12MAP and 15MAP). These garis were compared to the commercial cassava gari (CG).

The acidity (equivalent mg acetic acid/g) of the different cassava gari was significantly (*p* < 0.05) affected by the harvest period. This effect was well observed with the improved cassava varieties. Whatever the harvest period, gari from local cassava varieties exhibited the lowest value of acidity (Figure 2). The titratable acidity values of the gari samples were similar to those recommended by the Codex Alimentarius Commission [31].

Table 1 shows that the protein content of gari from cassava harvested at 12 months after planting (12MAP) exhibits high value (4.12–5.58% dry weight) compared to gari from cassava harvested at 15MAP. However, the commercial gari used as reference exhibited the lowest value for protein content. According to the harvest period, gari from local cassava varieties (*EN *and* AD*) were richer in protein than those from improved cassava varieties. But these differences were not observed with the free amino acid content of the gari (Table 1), whereas the amino acid content of the commercial gari was lower than that of the others gari whatever the harvest period and the variety. However, the same differences are not observed in the roots where those harvested at 12MAP showed the highest value of proteins (Table 1).

The lipids content of gari obtained from cassava harvested at 12MAP varied between 4.17 and 8.66%.  Table 2 shows that this range is higher than that of gari from cassava harvested at 15MAP. The commercial gari used as reference exhibited an intermediate value (5.43%). Gari from cassava harvested at 15MAP showed high value of fibres content (3.32–5.58%) than garis obtained from cassava harvested at 12MAP (3.05–4.74%). The phenolic compound content of the different gari samples obtained from cassava harvested at 12MAP varied significantly (*p* < 0.05) from 4.78 to 9.31 equivalent mg of gallic acid/g (dry weight) while those obtained from cassava harvested at 15MAP showed a range of 7.36 to 9.36 mg/g. It is observed in Table 2 that, from 15MAP to 12MAP, the phenolic compound content of local cassava gari (*EN* and* AD*) increases. However, these values decrease with the improved cassava gari.

**Figure 3:**
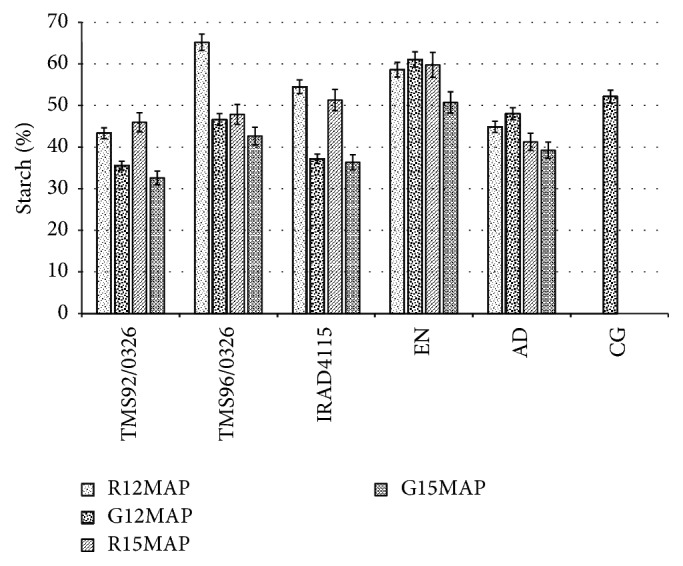
Effect of the harvest period (12 and 15 months after planting) on the starch content (g/100 g of dry weight) of cassava gari. Garis are produced from local (*EN *and* AD*) and improved (*TMS92/0326, TMS96/1414, *and* IRAD4115*) cassava varieties root harvested in dry season at 12 months and in rainy season at 15 months after planting (12MAP and 15MAP). These garis were compared to the commercial cassava gari (CG). G = gari and R = fresh root.

Gari from cassava harvested at 15MAP exhibited the highest value (3.47–9.71% dry weight) of soluble sugars (Table 2). Among these cassava gari that was obtained from local* EN* cassava variety was richer in free sugars than those from improved cassava varieties. The commercial cassava gari showed the lowest value of free sugars.  Figure 3 shows that the starch content of the cassava gari is significantly (*p* < 0.05) affected by the variety and the period of harvest. Starch content values ranged from 33 to 61% (dry weight). Whatever the variety, gari from cassava harvested at 12MAP exhibited the highest value of starch content. Gari from the* EN* local cassava variety was richer in starch than the other cassava garis. The total carbohydrates content (50 to 78%) of the cassava gari was also affected by the period of harvest and the variety. As for the starch content, it was also noticed that gari from cassava harvested at 12MAP shows the highest value (Table 2). The carbohydrates content of the commercial gari was close to those of the gari from cassava harvested at 15MAP in the rainy season.  Table 2 shows that gari from cassava harvested at 15MAP exhibits the highest value of crude fibres content (3.32–5.58%).

Table 3 shows that a fermentation process of two days reduced free cyanide content of the cassava root twofold to threefold. This reduction is well marked with gari from cassava harvested at 12MAP (0.08–0.74 mg HCN/g) compared to those from cassava harvested at 15MAP (0.51–0.90 mg HCN/g). However the commercial gari used as reference exhibited high value of free cyanide (2.03) as the cassava roots were dried before fermentation.

### 3.2. Functional Properties of Cassava Gari

The bulk density of gari from cassava harvested at 12MAP ranged from 0.52 to 0.62 g/ml (Table 3). This value reduced for gari produced from cassava harvested at 15MAP. Commercial gari and gari from local cassava gari* EN* exhibit the highest values for bulk density.

In Table 3, it is observed that the period of cassava harvest and the varietal differences affected the water absorption capacity (WAC) of gari. The WAC of the cassava gari varied from 4.49 to 8.02 ml/g. Whatever the variety, gari from cassava harvested at 12MAP showed the highest value of WAC. The WAC of commercial gari was closed to those of gari from cassava harvested at 15MAP. As for bulk density, gari from* EN* cassava harvested at 12MAP exhibits the highest value. In Table 3, similar observations were made with the swelling power of the different cassava gari.

The least gelatinization concentration (LGC) is the concentration at which gari slurry does not break down when tubes are inverted after a treatment at 90°C for one hour. The LGC of the different gari slurries differed significantly among varieties and for the two periods of harvest cassava root (Table 3). Gari from cassava harvested at 12MAP showed a LGC situated between 6.00 and 8.00% with the highest value recorded by the sample of TMS96/1414, IRAD4115, and* EN *cassava. These values were significantly (*p* < 0.05) lower than that of the commercial gari (14.00%).  Table 4 also shows that, from 12MAP to 15MAP, the least gelatinization concentration (LGC) increased.

**Figure 4:**
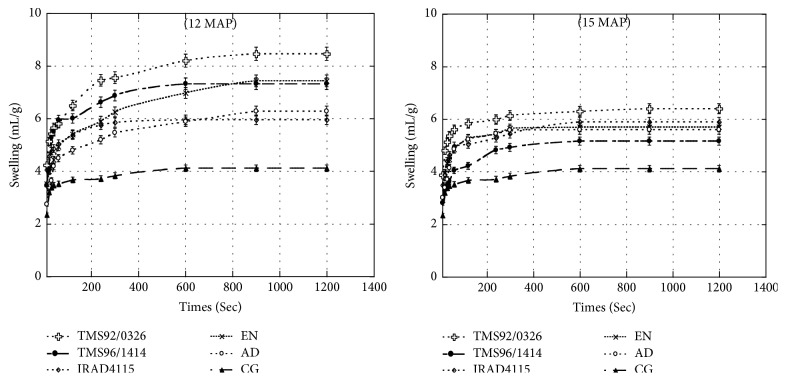
Effect of the harvest period (12 and 15 months after planting) on the swelling kinetic of cassava gari. Garis are produced from local (*EN* and* AD*) and improved (*TMS92/0326, TMS96/1414, *and* IRAD4115*) cassava varieties harvested in dry season at 12 months and in rainy season at 15 months after planting (12MAP and 15MAP). These garis were compared to the commercial cassava gari (CG).

As Figure 4 shows, the swelling kinetics of gari from cassava at 12MAP occurred in three stages. The first 100 seconds corresponded to the phase of fast swelling. This was followed by a slow swelling between 200 and 600 seconds depending on the gari sample. After this phase, a stationary phase of swelling took place. Gari from* TMS92/0326* cassava variety swelled faster than the other garis until stabilization and recorded the highest value for swelling over time. Although gari from* IRAD4115 *cassava swelled the least, it presented swelling kinetics higher than that of the commercial gari. There was a general reduction of swelling for all garis obtained from cassava harvested in the rainy season (at 15MAP). Gari from cassava harvested at 15MAP showed a similar trend; nevertheless gari from* TMS92/0326* and* EN *cassava varieties swelled faster between 10 and 250 seconds before decreasing until stabilization at 600 seconds (Figure 4).

### 3.3. Sensory Attributes of Cassava Gari

#### 3.3.1. Attributes of the Soaked Cassava Gari

Garis from cassava harvested at 12 and 15 months after planting (12MAP and 15MAP) were soaked into a sucrose solution (10%) and their sensory attributes were evaluated by 20 sensory trained panelists. 12MAP and 15MAP gari were served to the panelists in one session.  Table 4 shows that there was a significant (*p* < 0.05) difference between those attributes related to the variety of cassava and the harvest period. In terms of color, the panelists significantly (*p* < 0.05) preferred gari from cassava harvested at 12MAP. Gari from* EN *cassava had the best mark (4.50) followed by the commercial gari and gari from* AD *cassava (3.55). These two garis are from the root of local cassava varieties (Table 4). The odor of gari from cassava harvested at 12MAP was also well appreciated by the panelist compared to gari from cassava harvested at 15MAP. A similar appreciation was made for the commercial gari which also exhibited a good score for odor (3.15). As concerns the mouthfeel, it was observed that gari from cassava harvested at 15MAP showed the best score when compared to those from cassava harvested at 12MAP (Table 4). The commercial gari exhibited an intermediate score for mouthfeel. According to the panelists, garis from improved cassava varieties harvested at 12MAP were more acidic than those from cassava harvested at 15MAP. The effect of harvest period on mouthfeel was found not to be significant (*p* > 0.05) for gari from local cassava varieties. Gari from EN cassava harvested at 15MAP and the commercial gari were sweeter than the other garis among which no difference of sweetness was found.

In terms of overall acceptability, the panelists preferred gari from cassava harvested at 12MAP to gari from cassava harvested at 15MAP. The commercial gari used as reference had an intermediate score of preference. Concerning all the garis processed from cassava harvested at 12MAP (in the dry season), gari from local* EN* cassava variety had the best mark (4.30) followed by those from the improved* TMS92/0326 *and* TMS96/1414* cassava (3.75–3.80). The commercial gari showed an intermediate value of preference (3.55).

#### 3.3.2. Correlation between Organoleptic Attributes and the Preference of Panelists

For the determination of the effect of preference on the sensory interest of different gari samples on the score of the overall acceptability of panelists, a Chi-square test was carried out. Results showed that the overall acceptability of panelists for gari samples was significant and positively correlated with the color (*r* = +0.449; *p* < 0.01) and the odor (*r* = +0.380; *p* < 0.01). With the acidity and the sweetness, this correlation was nonsignificant. Moreover, the mouthfeel was significant and negatively correlated with the overall preference (*r* = −0.278; *p* < 0.01).

The general trend showed that gari samples processed at 12MAP were generally more acceptable for the consumers in terms of all sensory attributes while those obtained at 15MAP (rainy season) were the least preferred.

## 4. Discussion

### 4.1. Influence of Harvest Period and the Cassava Variety on the Proximate Composition of the Cassava Gari

The highest yield was observed with gari from cassava harvested during the dry season at 12 months after planting (12MAP). This may be attributed to factors such as plant age, varieties, and other environmental factors as mentioned by Oluwaniyi and Oladipo [32] and Wholey and Booth [33]. According to Adejumo and Raji [34], Sanni [35], and Karim et al. [36] most of carbohydrate in cassava harvested in the rainy season at 15MAP has been hydrolysed into free sugars. These differences in free sugars content are shown in Table 2. These sugars are used for growth of new plant tissues. The greater yield recorded by improved variety was also reported by Oghenechavwuko et al. [37] and may be attributed to a genetic factor.

The moisture content in the gari samples was lower than that of the commercial gari (16.60%). Indeed, it is reported that the high moisture content implies that a gari sample will not have a good storage potential [38], while the values obtained from gari samples of this study were good compared to those reported by Okolie et al. [39] for good storage potential and quality as well as those recommended by Oduro et al. [40]. The gari should be properly dried to a possible very low moisture content.

The cassava variety and the period of harvest had an effect on the pH of gari. Similar results were obtained by Egebebi and Aboloma [41], Kyereh et al. [42], and Nwafor et al. [43]. The acidic pH values of the cassava gari might have been due to the fermentation activity of microorganisms which used carbohydrate to produce more organic acids. The pH values of 4.6 to 5.8 recorded during this work are similar to those reported by Nwafor et al. [43] and by Ijabo and Igbo [44] with gari from local and improved varieties. The low pH value obtained with gari from cassava harvested at 12MAP could be due to the availability of carbohydrate to be metabolised by microorganism during the fermentation process. In particular free sugars found in the different cassava can favour growth of microorganisms in the mash. The pH and titratable acidity results of gari from improved cassava variety were well correlated. However, for the local cassava varieties, no significant difference was observed between garis from cassavas 12MAP and 15MAP. The titratable acidity values of the gari were similar to those observed by Bainbridge et al. [45] and almost as the values (0.60–1.0%) ranged by the Codex Alimentarius Commission for gari [31]. Plant age is a factor affecting the ash content of cassava roots [32]. The ash contents obtained in the present study were lower than those reported by Otutu et al. [46]. But these values were close to those of the Codex Standard for gari (1.5%) [31].

The high protein content of gari from cassava harvested at 12MAP could be linked to the high level of proteins in their corresponding roots (Table 1). This could also be due to the fact that, during the fermentation, microorganisms are more active in 12MAP cassava mash than in 15MAP cassava mash. In general, the local variety exhibited the highest values of protein content. However, cassava genetic is also a factor affecting the protein content. Oluwaniyi and Oladipo [32] obviously observed that the proteins content of TME 7 cassava variety root decreases from 7 to 12 months of age. When compared to commercial gari the difference could be due to the varietal differences. Additionally, in accord with Nwafor et al. [43], the ecological conditions can favour fermentation, mixed microorganisms involved to produce more amino acids which are used to synthesize proteins. According to Kobawila et al. [47], microorganisms can increase the crude protein content from 35 to 60% during fermentation depending on the quality of microorganisms involved in mash and the locality. The amino acids content of the different cassava gari is linked to the genetic differences. Moreover, the quality of microorganisms involved in mash fermentation could significantly influence the synthesis of amino acid or the breakdown of proteins. Probably the initial quantity of amino acids in storage roots may have an influence on the final quantity of amino acid in the gari (Table 1). The high quantity of amino acids observed in gari from cassava harvested at 12MAP could be attributed to the high temperature in the dry season, whence the water stress that confirm the significant effect of harvest period. In particular, it was reported that amino acids of storage roots increase during water stress [48, 49] which could be the case in the present study.

Since all the garis were prepared using the same amount of palm oil, differences of lipids content observed could be due to the varietal difference and the period of harvest. In the dry season (12MAP), cassava root might exhibit high lipid content compared to the rainy season (15MAP). This suggests that, in the rainy season, as with carbohydrate, lipid could also be hydrolysed to provide energy needed for root growth. This could be also due to genetic factors which affect plants to synthesize pretty much lipid in different environmental conditions. The values obtained in this study are slightly higher than those found (5.71% dry weight) by Onasoga et al. [50]. On the other hand, the crude lipid increase could be due to the activities of microorganisms during mash fermentation assuming conversion of carbohydrate into lipid and lipids for cellular growth. This was also reported by Padmaja et al. [51], Oboh and Akindahunsi [52], Fagbemi and Ijah [53], Boonnop et al. [54], and Ibukun and Anyasi [55]. The age of the cassava root used may favour the fibre content of the gari. This is why gari from cassava harvested at 15MAP exhibited higher value of fibre content. Since commercial gari showed similar values as gari obtained from 15MAP cassava root, it can be suggested that gari processed with old cassava root exhibits higher fibre content. Otutu et al. [46] reported that the values of crude fibre content of cassava gari ranged from 0.38 to 7.08% which were higher than those obtained in the present study. These results might have been due to removal of some fibre during gari sieving. Nevertheless, the values obtained in the present study are close to the value of crude fibre (2%) recommended by the Codex Standard [56].

The dry season favours hydrolysis of carbohydrates into free sugars. This confirms the significant effect of harvest period of cassava roots. Free sugars content is high in gari from improved cassava varieties. This could be due to genetic characteristics that fluctuate with the season and age of the plant [32]. The low value of free sugars found in the gari from cassava harvested at 15MAP may be due to the rainy season where free sugars are used for the new regrowth of leaves. The low value of sugar of commercial gari could suggest that the cassava roots were probably harvested in the rainy season.

The starch content of gari from cassava harvested at 12 months after planting (12MAP) was higher than that of gari from cassava harvested at 15MAP. In general the dry season (12 months after planting) is the period of the optimal starch storage in cassava than the rainy season. The highest value of starch content showed by gari from* EN *cassava variety could be due to its initial starch content in the storage roots (Figure 3). In this study, with the exception of gari from* EN *cassava, the starch contents of the other garis are similar to those of gari produced at six localities in Ghana [42]. The reduction in starch content in gari produced at 15MAP could be attributed to factors such as harvest season and plant age. In fact, gari produced from cassava harvested in the rainy season (15MAP) showed a reduction in starch content because of its mobilization for new shoot formation in the new growth cycle. This is in agreement with what was reported by Filho [57], Madore [58], Sriroth et al. [59], Sagrilo et al. [60], and Nuwamanya et al. [49]. Furthermore, cassava roots starch may decrease progressively as plant aged after optimal starch storage (Figure 3).

As shown in Table 2, the significantly high carbohydrates content showed by gari from* EN* cassava variety could be due to its initial carbohydrates content in the storage roots. The gari samples from* EN *cassava variety harvested at 12MAP showed significantly the highest value of carbohydrate than those from cassava harvested at 15 months as well as commercial gari. This might be due to varietal differences and harvest season as carbohydrate is highly concentrated during dry season compared to the rainy season. The reduction of carbohydrate for the gari samples obtained at 15 months was probably due to the rain which facilitates the hydrolysis of carbohydrate to sugar for shoots regrowth after prolonged water stress [60].

Certain cassava varieties produce more phenolic compounds when there is water stress than others justifying variation of phenolic compounds observed in this study. Gari from local* (EN)* and improved* (TMS96/1414)* cassava varieties presented significantly the same content in phenolic compounds when compared to commercial gari. This can be attributed to the processing method used. According to Etsuyankpa et al. [6] and Umezuruike et al. [61] roasting temperature and length of fermentation significantly decrease phenolic compounds.

The significant decrease in the level of residual cyanide observed in gari when compared to storage roots has been attributed to the degradation of cyanide (linamarin and lotaustralin) during cassava mash fermentation, dewatering, and roasting temperature. In fact, microorganisms involved in mash could have the ability to hydrolyse linamarin and lotaustralin. In addition, it was reported that heat significantly destroys cyanide in mash during roasting [11]. The slightly high levels of free cyanide from gari obtained at 15 months could be related to plant age and low activity of microorganisms involved in mash during the rainy season to significantly degrade cyanide into free cyanide (HCN). In fact, there is an optimum temperature for the optimum activity of any microorganisms to rapidly and completely break down the cyanogenic glucosides to cyanide acid. Gari from* TMS92/0326* improved cassava variety presented the least value of HCN. Regarding the initial storage roots cyanide content, the amount of cyanide acid in gari from local and improved cassava varieties was mostly dependent upon the processing method of garification. The highest level of free cyanide from commercial gari compared to gari produced may be due to the reduction in fermentation cassava mash to about 24 hours as well as quality of microorganisms present in mash.

### 4.2. Effect of Harvest Period and the Variety on the Functional Qualities of the Cassava Gari

The significant variation in bulk density (BD) may be attributed to varietal effect and climatic conditions [44, 62]. Nevertheless, the values of BD (0.47–0.49 g/ml) reported by Chika et al. [63] are lower than those observed in this study. However, bulk density obtained was lower than those reported by Olakunle et al. [64] and Oluwafemi and Udeh [7]; this could be due to fermentation length, moisture content, particles size; and processing method used. The highest value of BD recorded by* EN *gari from local variety could be correlated especially with the carbohydrates and starch content. During the dry roasting of the cassava mash, starch gelatinization occurs. This led to the formation of gari with different particles size. This suggests that gari with high particles size were from mash with high starch content.

Also, the gari samples differ in water absorption capacity (WAC). However, there was higher WAC from the gari produced at 12MAP compared to commercial gari but there was a reduction for those obtained at 15MAP. Similar results were observed by Chika et al. [63] though the values (3.58–4.17 g/g) were very low. However, these values were significantly higher when compared to some garis from three improved varieties reported by Nwancho et al. [62]. This may be attributed to size of the particles, garification length, and drying quality of gari. In fact, the WAC of gari decreases with decrease in particles size. Furthermore, a well dried gari samples should be able to absorb more water adequately when soaked compared to poorly dried gari samples.

The variation in swelling power among the gari samples when compared to commercial gari could be due to varietal factors and processing conditions used [44]. Yet we noted a general reduction in the swelling power for gari produced at 15MAP. These results can be attributed to the harvest of the storage root during the rainy season that lowered the amylose content responsible for great swelling power. In fact, all samples swelled as much as twice beyond their initial volume, which characterizes high quality gari produced from our cassava varieties [64, 65]. The variation in swelling power of gari from local and improved varieties could be explained by the amylose content of these garis.

The fluctuation of least gelation concentration (LGC) among garis when compared to commercial gari could be due to harvest season, fermentation time, and varietal differences. The highest value of LGC of commercial gari may be attributed to the size of particle and low amylose content. Furthermore, the LGC increased from 12MAP to 15MAP probably because of the decrease in roots amylase activity. In fact, the greater the percentage of amylose fraction of starch-based foods, the quicker the formation of the gel [66].

The differences linked to composition, particles size, fermentation length, and moisture contents [11, 64] could explain the significant variation of the swelling kinetics among gari samples. The* TMS96/0326* followed by* TMS96/1414* and* EN *gari samples exhibited the highest swelling ability and* IRAD4115 *gari sample the lowest for the gari produced at 12 months, while, at 15 months, the* IRAD4115* gari sample exhibited the highest swelling ability and* TMS96/1414* the least. The gari sample obtained at different time exhibited the best swelling ability when compared to commercial gari. These results may be related to varietal differences, the season, and the chemical compounds as well as processing method used.

### 4.3. Effect of the Harvest Period and the Variety on Sensory Attribute of the Cassava Gari

A significant difference was noted between gari samples produced at 12 months and 15 months and with commercial gari. The differences observed in the color and taste could be due to the fact that different processing methods were used during production of gari samples, especially the length of fermentation which might differ from one locality to the other. Moreover, each of the mixtures of microorganism involved in mash fermentation has its effect on sensory quality as stated by Olaoye et al. [67]. It had been observed that panelists appreciated gari samples quality based on color or appearance and odor. The result is in agreement with Agbor-Egbe and Mbome [12], who reported that gari preference was attributed to the better organoleptic characteristics such as appearance, odor, particle size, swelling properties, and eating quality. This is confirmed by a significant positive correlation noted between color, odor, and preference (*r* = +0.449; *p* < 0.01 and *r* = +0.380; *p* < 0.01, resp.). The gari sample from* EN* obtained at 12 months was significantly preferred in terms of general acceptability than those produced at 15 months. This could be due to rainy season which affects the color and odor. Similarly, Agbor-Egbe and Mbome [12] observed that the gari produced using the local cassava variety was more preferred than those from the improved variety. Additionally, the choice of* EN *gari was correlated to the local sweet and red cassava used for the garification. This is in agreement with Olaoye et al. [67].

## 5. Conclusion

This study shows that the gari yield from* TMS96/1414 *(improved variety) is 63.72% and 55.91% produced in dry season at 12 months and in rainy season at 15 months, respectively, followed by those from* EN* (local variety). The study reveals that age, season at which cassava storage roots are harvested, and variety affects some physicochemical, functional, and sensory properties of gari. The study also shows that processing cassava roots into gari significantly reduces the cyanide acid (HCN) at level considered safe. In addition, gari samples produced in the dry season are better in terms of physicochemical, functional, and sensory properties than gari produced in the rainy season. The general trends show that gari samples processed in the dry season (12MAP) were generally acceptable to the consumers in terms of all sensory attributes while those obtained in the rainy season (15MAP) and commercial gari were the least preferred. In the far north region of Cameroon, all cassava roots varieties can be processed into gari, but* EN* (red, sweet),* TMS92/0326,* and* TMS92/1414* can be processed into gari with the best physicochemical, functional, and sensory properties compared to commercial gari. Thus, quality gari can contribute to overcoming malnutrition and undernourishment in the far north region in particular and the whole of Cameroon in general.

## Figures and Tables

**Table 1:** Impact of the harvest period (12 and 15 months after planting) on the pH, the dry matter, the ash, the crude proteins, and the free amino acids content of cassava gari. Garis are produced from local (EN and AD) and improved (TMS92/0326, TMS96/1414, and IRAD4115) cassava varieties root harvested in dry season at 12 months and in rainy season at 15 months after planting (12MAP and 15MAP). These garis were compared to the commercial cassava gari (CG). Values are given on the dry weight basis.

Harvest periods and varieties		Dry matter (%)		Ash		pH	Proteins (%)		Amino acids (mg/g)	
		Gari	Root	Gari (mg/g)	Root (mg/g)	Gari	Gari	Root	Gari	Root
12MAP (May)	*TMS92/0326*	96.60 ± 0.37^c^	25.21 ± 0.32^d^	20.61 ± 0.80^a^	8.55 ± 0.19^c^	4.31 ± 0.14^c^	4.12 ± 0.15^c^	6.79 ± 0.34^d^	15.51 ± 0.45^b^	11.25 ± 0.84^c^
	*TMS96/1414*	97.30 ± 0.03^a^	28.39 ± 0.33^b^	19.59 ± 0.71^d^	8.64 ± 0.33^c^	4.58 ± 0.22^cdb^	5.03 ± 0.16^b^	9.75 ± 0.41^a^	14.82 ± 0.38^c^	12.16 ± 0.22^a^
	*IRAD4115*	97.28 ± 0.11^ab^	25.55 ± 0.46^d^	20.62 ± 1.19^d^	8.35 ± 0.18^c^	4.72 ± 0.25^d^	4.17 ± 0.09^c^	7.40 ± 0.21^c^	16.72 ± 0.29^a^	14.98 ± 0.48^b^
	*EN*	96.91 ± 0.09^bc^	30.25 ± 0.27^a^	19.80 ± 0.36^d^	8.50 ± 0.14^c^	4.84 ± 0.27^bd^	5.58 ± 0.20^a^	5.86 ± 0.19^ef^	11.97 ± 0.41^f^	10.21 ± 0.33^h^
	*AD*	96.80 ± 0.18^bc^	25.66 ± 0.39^d^	22.30 ± 1.47^bc^	7.79 ± 0.25^d^	4.50 ± 0.19^cdb^	5.40 ± 0.18^a^	5.90 ± 0.36^ef^	14.07 ± 0.24^d^	8.91 ± 0.28^g^

15MAP (August)	*TMS92/0326*	96.60 ± 0.37^c^	22.23 ± 0.09^e^	24.16 ± 0.56^a^	10.14 ± 0.25^b^	5.28 ± 0.04^b^	2.72 ± 0.14^d^	5.48 ± 0.33^f^	12.07 ± 0.19^f^	7.82 ± 0.32^f^
	*TMS96/1414*	96.52 ± 0.07^c^	25.72 ± 0.46^cd^	23.36 ± 0.72^ab^	10.57 ± 0.18^b^	5.29 ± 0.02^b^	2.08 ± 0.08^ef^	8.93 ± 0.25^b^	14.95 ± 0.31^bc^	7.83 ± 0.16^d^
	*IRAD4115*	96.60 ± 0.83^c^	22.26 ± 0.12^e^	24.02 ± 0.47^a^	11.25 ± 0.48^a^	5.28 ± 0.05^b^	2.28 ± 0.11^e^	6.33 ± 0.35^de^	12.59 ± 0.28^ef^	7.21 ± 0.17^i^
	*EN*	96.40 ± 0.06^c^	26.31 ± 0.28^c^	20.37 ± 0.58^d^	10.80 ± 0.51^ab^	5.30 ± 0.04^b^	5.58 ± 0.18^a^	6.05 ± 0.17^de^	13.96 ± 0.45^d^	9.44 ± 0.28^j^
	*AD*	96.31 ± 0.05^c^	22.38 ± 0.12^e^	24.49 ± 0.74^a^	10.70 ± 0.39^ab^	5.17 ± 0.05^c^	4.43 ± 0.12^c^	6.61 ± 0.31^d^	12.94 ± 0.33^e^	6.53 ± 0.37^k^

Commercial cassava gari (CG)		83.48 ± 0.28^d^	ND	21.78 ± 0.35^c^	ND	5.43 ± 0.03^a^	2.00 ± 0.08^f^	ND	6.04 ± 0.13^g^	ND

Values are means ± standard deviation of triplicates (*n* = 3). Values in the same column with the different superscript are significantly different (*p* < 0.05).

**Table 2:** Effect of the harvest period (12 and 15 months after planting) on the phenolic compounds (PC), the total carbohydrates, the free sugars, the fibre, and the lipids content of cassava gari. Gari are produced from local (EN and AD) and improved (TMS92/0326, TMS96/1414, and IRAD4115) cassava varieties root harvested in dry season at 12 months and in rainy season at 15 months after planting (12MAP and 15MAP). These garis were compared to the commercial cassava gari (CG). Values are given on the dry weight basis.

Harvest periods and varieties		Characteristics							
		PC (Equivalent mg gallic acid/g)	Carbohydrates (%)		Sugars (%)		Fibres (%)		Lipids (%)
			Gari	Root	Gari	Root	Gari	Root	
12MAP (May)	*TMS92/0326*	8.63 ± 0.16^bc^	75.12 ± 0.57^b^	86.14 ± 2.01^b^	9.71 ± 0.10^a^	25.45 ± 0.55^b^	4.43 ± 0.10^e^	3.70 ± 0.10^c^	5.85 ± 0.14^c^
	*TMS96/1414*	9.31 ± 0.21^a^	65.98 ± 0.84^e^	74.54 ± 3.26^d^	6.18 ± 0.06^b^	20.77 ± 0.62^c^	3.70 ± 0.08^g^	3.05 ± 0.18^de^	8.66 ± 0.26^a^
	*IRAD4115*	7.43 ± 0.31^de^	71.68 ± 0.99^c^	79.36 ± 2.48^c^	4.57 ± 0.07^e^	15.30 ± 0.47^e^	3.05 ± 0.06^i^	2.89 ± 0.11	6.66 ± 0.12^b^
	*EN*	8.01 ± 0.26^cd^	78.07 ± 0.87^a^	90.15 ± 3.25^a^	3.47 ± 0.06^f^	15.68 ± 0.41^e^	4.74 ± 0.05^c^	3.24 ± 0.14^d^	4.17 ± 0.33^f^
	*AD*	4.78 ± 0.19^g^	62.62 ± 1.29^f^	74.61 ± 2.18^d^	5.66 ± 0.09^c^	17.17 ± 0.37^d^	4.07 ± 0.07^f^	2.88 ± 0.10^e^	6.45 ± 0.25^b^

15MAP (August)	*TMS92/0326*	7.36 ± 0.11^e^	58.73 ± 1.29^g^	82.25 ± 2.16^bc^	5.04 ± 0.05^d^	30.80 ± 0.48^a^	5.58 ± 0.05^a^	5.01 ± 0.16^a^	3.93 ± 0.12^fg^
	*TMS96/1414*	7.36 ± 0.16^e^	52.62 ± 0.26^i^	71.25 ± 2.04^e^	3.21 ± 0.11^g^	29.87 ± 0.49^a^	4.44 ± 0.13^e^	4.11 ± 0.13^b^	5.22 ± 0.29^e^
	*IRAD4115*	7.46 ± 0.07^e^	49.58 ± 0.95^j^	75.24 ± 2.43^d^	3.44 ± 0.09^f^	19.47 ± 0.27^c^	3.32 ± 0.11^h^	3.01 ± 0.11^e^	4.18 ± 0.26^f^
	*EN*	9.36 ± 0.10^a^	68.47 ± 0.92^d^	86.57 ± 2.37^b^	4.56 ± 0.05^e^	20.83 ± 0.72^c^	5.10 ± 0.07^b^	4.05 ± 0.14^b^	2.66 ± 0.32^h^
	*AD*	8.74 ± 0.28^b^	54.72 ± 1.37^hi^	72.34 ± 1.87^de^	5.16 ± 0.08^d^	19.71 ± 0.53^c^	4.58 ± 0.11^de^	4.04 ± 0.12^b^	3.61 ± 0.24^g^

Commercial cassava gari (CG)		6.01 ± 0.16^f^	56.84 ± 0.71^gh^	ND	2.45 ± 0.08^h^	ND	4.62 ± 0.09^cd^	ND	5.43 ± 0.03^d^

Values are means ± standard deviation of triplicates (*n* = 3). Values in the same column with the different superscript are significantly different (*p* < 0.05).

**Table 3:** Effect of the harvest period (12 and 15 months after planting) on the bulk density, the water absorption capacity (WAC), the swelling power, the least gelation concentration and the free cyanide content of cassava gari. Gari are produced from local (EN and AD) and improved (TMS92/0326, TMS96/1414 and IRAD4115) cassava varieties harvested in dry season at 12 months and in rainy season at 15 months after planting (12MAP and 15MAP). These garis were compared to the commercial cassava gari (CG). Values are given on the dry weight basis.

Harvest periods	Varieties	Free Cyanide (Equivalent mg HCN/g)		Bulk density (g/mL)	WAC (g/g)	Swelling power (mL/g)	Least gelation concentration (%)
		Storage roots	Gari				
12MAP (May)	*TMS92/0326*	1.47 ± 0.04^e^	0.08 ± 0.01^g^	0.56 ± 0.02^bc^	7.99 ± 0.06^a^	8.37 ± 0.07^b^	8.00
	*TMS96/1414*	1.56 ± 0.06^cd^	0.23 ± 0.04^f^	0.55 ± 0.02^c^	6.58 ± 0.08^b^	7.54 ± 0.11^c^	8.00
	*IRAD4115*	1.20 ± 0.05^f^	0.74 ± 0.03^c^	0.52 ± 0.01^d^	5.29 ± 0.07^e^	7.24 ± 0.08^d^	6.00
	*EN*	0.88 ± 0.03^g^	0.57 ± 0.05^a^	0.62 ± 0.01^a^	8.02 ± 0.09^a^	8.86 ± 0.11^a^	6.00
	*AD*	1.18 ± 0.05^f^	0.72 ± 0.08^cd^	0.57 ± 0.01^b^	6.79 ± 0.20^b^	7.52 ± 0.05^c^	6.00

15MAP (August)	*TMS92/0326*	2.52 ± 0.09^a^	0.67 ± 0.02^d^	0.47 ± 0.02^e^	5.78 ± 0.04^d^	6.77 ± 0.09^f^	9.00
	*TMS96/1414*	1.54 ± 0.05^de^	0.67 ± 0.05^d^	0.50 ± 0.01^e^	5.94 ± 0.09^c^	6.94 ± 0.13^e^	9.00
	*IRAD4115*	1.67 ± 0.09^c^	0.51 ± 0.06^e^	0.48 ± 0.03^e^	5.05 ± 0.11^f^	6.62 ± 0.12^f^	10.00
	*EN*	1.49 ± 0.05^e^	0.59 ± 0.05^e^	0.53 ± 0.02^cd^	4.49 ± 0.08^g^	5.53 ± 0.06^h^	9.00
	*AD*	1.85 ± 0.06^b^	0.90 ± 0.08^b^	0.51 ± 0.03^de^	4.50 ± 0.06^g^	5.44 ± 0.08^h^	9.00

Commercial cassava gari (CG)		ND	2.03 ± 0.04^a^	0.61 ± 0.02^a^	5.37 ± 0.07^e^	6.35 ± 0.04^g^	14.00

Values are means ± standard deviation of triplicates (*n* = 3). Values in the same column with the different superscript are significantly different (*p* < 0.05).

**Table 4:** Hedonic sensory mean scores of cassava gari. Garis are produced from local (EN and AD) and improved (TMS92/0326, TMS96/1414, and IRAD4115) cassava varieties harvested in dry season at 12 months and in rainy season at 15 months after planting (12MAP and 15MAP). These garis were compared to the commercial cassava gari (CG).

Harvest periods	Varieties	Color	Odor	Mouthfeel	Acidity	Sweetness	Overall acceptability
12MAP (May)	*TMS92/0326*	3.45 ± 0.69^bc^	3.40 ± 0.82^a^	2.55 ± 0.51^e^	2.80 ± 0.77^a^	2.55 ± 0.51^bcd^	3.75 ± 0.72^b^
	*TMS96/1414*	2.85 ± 0.67^d^	3.20 ± 0.89^a^	2.95 ± 0.69^cde^	2.65 ± 0.75^ab^	2.65 ± 0.75^bc^	3.80 ± 0.69^b^
	*IRAD4115*	2.75 ± 0.85^d^	3.05 ± 0.89^ab^	3.30 ± 0.57^bc^	2.20 ± 0.41^bc^	2.50 ± 0.95^bcd^	2.75 ± 0.91^de^
	*EN*	4.50 ± 0.76^a^	3.50 ± 0.76^a^	2.75 ± 0.85^de^	1.60 ± 0.50^d^	2.60 ± 0.68^bcd^	4.30 ± 0.81^a^
	*AD*	3.55 ± 0.78^bc^	2.95 ± 0.94^ab^	1.90 ± 0.71^f^	1.65 ± 0.75^d^	2.30 ± 0.75^cd^	3.10 ± 0.79^cd^

15MAP (August)	*TMS92/0326*	2.50 ± 0.69^de^	1.80 ± 0.83^d^	4.30 ± 0.86^a^	1.90 ± 0.85^cd^	2.30 ± 0.86^cd^	2.55 ± 0.69^e^
	*TMS96/1414*	2.15 ± 0.81^e^	2.50 ± 0.60^bc^	3.25 ± 0.79^bc^	2.00 ± 1.03^cd^	2.25 ± 0.78^cd^	2.75 ± 0.79^de^
	*IRAD4115*	2.40 ± 0.75^de^	2.35 ± 0.49^c^	3.15 ± 0.75^bcd^	1.85 ± 0.75^cd^	3.00 ± 0.79^ab^	2.40 ± 0.50^e^
	*EN*	2.20 ± 0.41^e^	2.55 ± 0.83^bc^	3.60 ± 0.75^b^	1.95 ± 0.89^cd^	3.35 ± 0.99^a^	2.50 ± 0.76^e^
	*AD*	3.40 ± 0.68^c^	2.95 ± 0.94^ab^	1.85 ± 0.49^f^	1.65 ± 0.75^d^	2.05 ± 0.76^d^	3.00 ± 0.83^cd^

Commercial gari cassava (CG)		3.90 ± 0.55^b^	3.15 ± 0.93^a^	2.85 ± 0.81^cde^	2.50 ± 0.69^ab^	2.95 ± 0.60^ab^	3.55 ± 0.51^bc^

Values are means ± standard deviation of scores awarded by panelists (*n* = 20). Values in the same column with the different superscript are significantly different (*p* < 0.05).

## References

[1] Ukwuru M. U., E Egbonu S. (2013). Recent development in cassava-based products research. *Academia Journal of Food Research*.

[2] Ola L., Rihana H., Siew N. (2016). Identification of aromatic compounds and their sensory characteristics in cassava flakes and garri (Manihot esculenta Crantz, CyTA). *Journal of Food*.

[3] Mduduzi P. M., Taurai M., Ademola O. O. (2016). Perspectives on the probiotic potential of lactic acid bacteria from African traditional fermented foods and beverages. *Food and nutrition Research*.

[4] Oyeyipo O. O. (2011). *Studies on Lafun Fortified with African breadfruit Tempeh [Msc. thesis]*.

[5] Oboh G. (2006). Nutrient enrichment of cassava peels using a mixed culture of Saccharomyces cerevisiae and Lactobacillus spp solid media fermentation techniques. *Electronic Journal of Biotechnology*.

[6] Etsuyankpa M. B., Gimba C. E., Agbaji E. B., Omoniyi I., Ndamitso M. M., Mathew J. T. (2015). Assessment of the Effects of Microbial Fermentation on Selected Anti-Nutrients in the Products of Four Local Cassava Varieties from Niger State, Nigeria. *American Journal of Food Science and Technology*.

[7] Oluwafemi G. I., Udeh C. C. (2016). Effect of fermentation periods on the physicochemical and sensory properties of gari. *Journal of Environmental Science*.

[8] Achinewhu S. C., Barber L. I., Ijeoma I. O. (1998). Physicochemical properties and garification (gari yield) of selected cassava cultivars in Rivers State, Nigeria. *Plant Foods for Human Nutrition*.

[9] Tetchi F. A., Solomon O. W., Celah K. A., George A. N. (2012). Effect of cassava variety and fermentation time on biochemical and microbiological characteristics of raw artisanal starter for Attieke production. *Innovative Romanian Food Biotechnology*.

[10] Mahungwu N. M., Yamaguchi Y., Almazan A. M., Hatin S. K. (1987). Reduction of cyanide during processing of cyanide of cassava into some traditional African foods. *Journal of Food and Agriculture*.

[11] Irtwange S. V., Achimba O. (2009). Effect of the Duration of Fermentation on the Quality of Gari. *Current Research Journal of Biological Science*.

[12] Agbor-Egbe T., Mbome I. L. (2006). The effects of processing techniques in reducing cyanogen levels during the production of some Cameroonian cassava foods. *Journal of Food Composition and Analysis*.

[13] Nweke F. I., Spencer D. S. C., Lynam J. K. (2002). *The cassava transformation: Africa’s best kept secret*.

[14] Babalola O. O. (2014). Cyanide Content of Commercial Gari from Different Areas of Ekiti State, Nigeria. *World Journal of Nutrition and Health*.

[15] Halliday D., Qureshi H. A., Broadbent J. A. (1967). Investigation on the storage of gari Nig. Stored Prod. *Research Institute Technical Report*.

[16] Amamgbo L. E. F., Akinpelu A. O., Omodamiro R., Nwakor F. N., Ekedo T. O. (2016). Promotion and popularization of some elite cassava varieties in Igbariam Anambra State: implication for food security and smpowerment. *Global Advanced Research Journal of Agricultural Science*.

[17] Sobowale S. S., Awonorin S. O., Shittu T. A., Oke M. O., Adebo O. A. (2016). Estimation of material losses and the effects of cassava at different maturity stages on garification index. *Journal of Food Processing and Technology*.

[18] Herwitz W., AOAC (1990). Official methods of analysis of AOAC International. *Association of Official Analytical Chemists*.

[19] Devani M. B., Shishoo C. J., Shal S. A., Suhagia B. N. (1989). Spectrophotometric method for microdetermination of nitrogen in Kjedahl digest. *Journal of Association Official Analytical Chemists*.

[20] AFNOR (Association Française pour la Normalisation) (1984). Produits alimentaires : directives générales pour le dosage de l’azote avec minéralisation selon la méthode de kjedahl. *Godon et Pineau*.

[21] Michel M. C. (1968). Dosage des acides aminés et amines par la ninhydrine. Amélioration pratique, Annal de Biologie Animale. *Biochimie, Biophysique*.

[22] Tollier M. T., Robin J. P. (1979). Adaptation of the method sulfuric orcinol automatic determination of total neutral carbohydrates. Terms of adaptation to extracts of vegetable origin. *Annals of Technological Agriculture*.

[23] Jarvis C. E., Walker J. R. L. (1993). Simultaneous, rapid; spectrophotometric determination of total starch, amylase and amylopectine. *Journal of the Science of Food and Agriculture*.

[24] Singleton V. L., Orthofer R., Lamuela-Raventós R. M. (1999). Analysis of total phenols and other oxidation substrates and antioxidants by means of folin-ciocalteu reagent. *Methods in Enzymology*.

[25] de B. Baltha A. D. T., Cereda M. P. Cassava free cyanide analysis using KCN or acetone-cyanidrin as pattern.

[26] Adeleke R. O., Odedeji J. O. (2010). Functional properties of wheat and sweet potato flour blends. *Pakistan Journal of Nutrition*.

[27] Koubala B. B., Kansci G., Enone E. L. B., Dabole N. O., Yaya N. V., Zang E. M. A. (2014). Effect of Fermentation Time on the Physicochemical and Sensorial Properties of Gari from Sweet Potato (Ipomoae batatas). *British Journal of Applied Science and Technology*.

[28] Hung P. V., Maeda T., Morita N. (2007). Dough and bread qualities of flours with whole waxy wheat flour substitution. *Food Research International*.

[29] Nnanna H. A., Phillips R. D., McWatters K. H., Hung Y.-C. (2002). Effect of germination on the physical, chemical, and sensory characteristics of cowpea products: Flour, paste, and akara. *Journal of Agricultural and Food Chemistry*.

[30] AFNOR (1980). *Collection of French Standards of General Methods of Analysis of Food Products: Chemistry, Microbiology, Sensory Analysis*.

[31] Codex Alimentarius Commission (1989). Codex Standard for Gari. *Codex Stan 151-1989 (Rev, 1-1995)*.

[32] Oluwaniyi O., Oladipo J. (2017). Comparative studies on the phytochemicals, nutrients and antinutrients content of cassava varieties. *Journal of the Turkish Chemical Society, Section A: Chemistry*.

[33] Wholey D. W., Booth R. H. (1979). Influence of variety and planting density on starch accumulation in cassava roots. *Journal of the Science of Food and Agriculture*.

[34] Adejumo B. A., Raji A. O. (2010). An appraisal of gari packaging in Ogbomoso, Southwestern Nigeria. *Journal of Agricultural and Veterinary Sciences*.

[35] Sanni L. O. (1990). Hazard analysis of critical control points in the commercial production of high quality gari. *Nigeria Journal of Science*.

[36] Karim O. R., Fasasi O. S., Oyeyinka S. A. (2009). Gari yield and chemical composition of cassava roots stored using traditional methods. *Pakistan Journal of Nutrition*.

[37] Oghenechavwuko U. E., Olasunkanmi S. G., Adekunbi T. K., Taiwo A. C. (2013). Effect of processing on the physico-chemical properties and yield of gari from dried chips. *Journal of Food Processing and Technology*.

[38] Ukenye E., Ukpabi U. J., Chijoke U., Egesi C., Njoku S. (2013). Physicochemical, nutritional and processing properties of promising newly bred white and yellow fleshed cassava genotypes in Nigeria. *Pakistan Journal of Nutrition*.

[39] Okolie N. P., Brai M. N., Atotebi O. M. (2012). Comparative study on some selected garri samples sold in lagos metropolis. *Journal of Food Studies*.

[40] Oduro I., Ellis W. O., Dziedzoave N. T., Nimako-Yeboah K. (2000). Quality of gari from selected processing zones in Ghana. *Food Control*.

[41] Egebebi A. O., Aboloma R. I. (2012). Fungi and moisture content of gari sold in some locations in Southwestern Nigeria. *Archives of Applied Science Research*.

[42] Kyereh E., Bani R. J., Obeng-Ofori D. (2013). Effect of cassava processing equipment on quality of gari produce in selected processing site in Ghana. *International Journal of Agriculture Innovations and Research*.

[43] Nwafor O. E., Akpomie O. O., Erijo P. E. (2015). Effect of fermentation time on the physico-chemical, nutritional and sensory quality of cassava chips (Kpo-Kpo garri) a traditional nigerian food. *American Journal of BioScience*.

[44] Ijabo O. J., Igbo P. K. (2016). Effects of three lower benue grown cassava (Manihot esculanta) varieties and processes on seven quality indices of fresh gari. *International Journal of Engineering and Advanced Research Technology*.

[45] Bainbridge Z., Tomlins K., Welling K., Westby A. (1996). *Methods for Assessing Quality Characteristics of Non-Grain Starch Staple (Part 2. Filed Methods)*.

[46] Otutu O. L., Ikuomola D. S., Udom Q. (2013). Comparative evaluation of quality of gari samples from six processing centres in oriade lga of osun state, Nigeria. *IJAFS*.

[47] Kobawila S. C., Louembe D., Keleke S., Hounhouigan J., Gamba C. (2005). Reduction of the cyanide content during fermentation of cassava roots and leaves to produce bikedi and ntoba mbodi, two food products from Congo. *African Journal of Biotechnology*.

[48] Rodrigues B. M., Souza B. D., Nogueira R. M., Santos M. G. (2010). Tolerance to water deficit in young trees of jackfruit and sugar apple. *Revista Ciência Agronômica*.

[49] Nuwamanya E., Rubaihayo P. R., Mukasa S., Kyamanywa S., Hawumba J. F., Baguma Y. (2014). Biochemical and secondary metabolites changes under moisture and temperature stress in cassava (Manihot esculenta Crantz). *African Journal of Biotechnology*.

[50] Onasoga M., Oluwafunmilayo D., Oluwafunmilayo., Oyeyipo O. O. (2014). Chemical Changes during the Fortification of Cassava Meal (Gari) with African breadfruit (Treculia africana) Residue. *Journal of Applied Science and Environ Management*.

[51] Padmaja G., George M., Moorthy S. N. (1993). Detoxification of cassava during fermentation with a mixed culture inoculum. *Journal of the Science of Food and Agriculture*.

[52] Oboh G., Akindahunsi A. A. (2003). Biochemical changes in cassava products (flour & gari) subjected to Saccharomyces cerevisae solid media fermentation. *Food Chemistry*.

[53] Fagbemi A. O., Ijah U. J. J. (2006). Microbial population and biochemical changes during production of protein-enriched fufu. *World Journal of Microbiology and Biotechnology*.

[54] Boonnop K., Wanapat M., Nontaso N., Wanapat S. (2009). Enriching nutritive value of cassava root by yeast fermentation. *Scientia Agricola*.

[55] Ibukun E. O., Anyasi O. J. (2013). Changes in antinutrient and nutritional values of fermented sesame (Sesanum indicum), Musk Melon (Cucumis melo) and white melon (Cucumeropsis mannii). *International Journal of Advanced Biotechnology and Research*.

[56] Sanni L. B., Maziya-Dixon J., Akanya C. I., Okoro Y., Alaya C. V., Egwuonwu R. (2005). *Standards for Cassava Poducts and Gidelines for Export*.

[57] Filho S. J. B. (1980). *Distribuição de carboidratos em plantas de mandioca (Manihot esculenta, Crantz) e o efeito do teor de reservas, na brotação e enraizamento de estacas de três posições do caule*.

[58] Madore M. A., Pessorakli M. (1994). Phloem transport of solutes in crop plants. *Handbook of Plant and Crop Physiology*.

[59] Sriroth K., Piyachomkwan K., Santisopasri V., Oates C. G. (2001). Environmental conditions during root development: Drought constraint on cassava starch quality. *Euphytica*.

[60] Sagrilo E., Vidigal Filho P. S., Pequeno M. G. (2003). Effect of harvest period on the quality of storage roots and protein content of the leaves in five cassava cultivars (Manihot esculenta, Crantz). *Brazilian Archives of Biology and Technology*.

[61] Umezuruike A. C., Nwabueze T. U., Akobundu E. N. T. (2016). Anti nutrient content of food grade flour or roasted African breadfruit seeds produced under extreme condition. *Academia Journal of Biotechnology*.

[62] Nwancho S. O., Ekwu F. C., Mgbebu P. O., Njoku C. K., Okoro C. (2014). Effect of particle size on the functional, pasting and textural properties of gari produced from fresh cassava roots and dry chips. *The International Journal of Engineering and Science*.

[63] Chika C., Ogueke C. E., Owuamanam C. I., Ahaotu I., Olawuni I. (2013). Quality characteristics and HCN in gari as affected by fermentation variables. *International Journal of Life Sciences*.

[64] Olakunle M. M., Akinwale S. O., Makanjuola J. O., Awonorin S. O. (2012). Comparative study on quality attributes of gari obtained from some processing centers in South West, Nigeria. *Advance Journal of Food Science and Technology*.

[65] Sanni L. A., Odukogbe O. O., Faborode M. O., Ibrahim R. O. (2015). Optimization of process parameters of a conductive rotary dryer for gari production. *Journal of Emerging Trends in Engineering and Applied Sciences*.

[66] Sanni L. O., Kosoko S. B., Adebowale A. A., Adeoye R. J. (2004). The influence of palm oil and chemical modification on the pasting and sensory properties of fufu flour. *International Journal of Food Properties*.

[67] Olaoye O. A., Lawrence I. G., Cornelius G. N., Ihenetu M. E. (2015). Evaluation of quality attributes of cassava product (gari) produced at varying length of fermentation. *American Journal of Agricultural Science*.

